# Integrated Stress Response (ISR) Modulators in Vascular Diseases

**DOI:** 10.3390/cells15010002

**Published:** 2025-12-19

**Authors:** Alexander Kalinin, Ekaterina Zubkova, Irina Beloglazova, Yelena Parfyonova, Mikhail Menshikov

**Affiliations:** 1 National Medical Research Center of Cardiology Named after Academician E.I. Chazov, Ministry of Health of the Russian Federation, Moscow 121552, Russia; 2 Department of Biochemistry and Regenerative Biomedicine, Faculty of Fundamental Medicine, Lomonosov Moscow State University, Moscow 119991, Russia

**Keywords:** integrated stress response, pulmonary arterial hypertension, pulmonary veno-occlusive disease, thrombosis, restenosis, pulmonary capillary hemangiomatosis, PERK, PKR, GCN2, cardiovascular diseases

## Abstract

**Highlights:**

**What are the main findings?**

- The integrated stress response (ISR) has emerged as an important regulator of vascular homeostasis and pathology, orchestrating endothelial adaptation to metabolic, oxidative, and inflammatory stress through coordinated translational and transcriptional control.
- PERK and PKR signaling pathways promote maladaptive vascular remodeling under chronic stress, driving endothelial apoptosis, inflammation, and pathological neovascularization, whereas GCN2 exerts protective effects, particularly in the pulmonary circulation.

**What are the implications of the main findings?**

- Selective pharmacological targeting of ISR components offers therapeutic promise, with both inhibitors (e.g., GSK2606414, 2-aminopurine, C16) and activators (e.g., salubrinal, halofuginone) demonstrating efficacy in preclinical models of atherosclerosis, restenosis, thrombosis, and pulmonary hypertension.
- The dual and context-dependent roles of ISR signaling underscore the need for precision-targeted modulation, with therapeutic outcomes varying by disease state, cellular context, and the specific ISR kinase engaged.
- Translational progress is currently limited by gaps in pharmacokinetics, selectivity, and long-term safety profiles of ISR modulators, highlighting the necessity for mechanistic dissection and in vivo validation to support clinical application.

**Abstract:**

Vascular dysfunction lies at the core of cardiovascular diseases—the leading cause of global morbidity and mortality. Despite their prevalence, therapeutic options remain limited, in part due to an incomplete understanding of the molecular mechanisms driving vascular pathology. The integrated stress response (ISR), an evolutionarily conserved signaling network activated by diverse stressors, represents a critical but underexplored mechanism in vascular biology. This review examines the dual roles of the core ISR kinases—PERK, GCN2, HRI and PKR—in vascular homeostasis and pathology, including atherosclerosis, pulmonary hypertension, and angiogenesis. We develop a conceptual framework in which the ISR functions as a context-dependent, double-edged sword: while PERK and PKR promote inflammation, apoptosis, and vascular re-modeling, GCN2 mediates protective effects. The outcome of ISR activation is shaped by cell type, stress duration and intensity, and downstream signaling bias (e.g., ATF4 vs. CHOP dominance). We further discuss pharmacological ISR modulators—including 2-aminopurine, C16, salubrinal, halofuginone, GSK2606414, and GSK2656157—which have demonstrated beneficial effects in preclinical models by suppressing inflammation, reducing apoptosis, and attenuating disease progression. Collectively, the ISR emerges as a critical regulatory node in vascular pathophysiology, and its selective, context-aware modulation represents a promising avenue for therapeutic intervention.

## 1. Introduction

The integrated stress response (ISR) is an evolutionarily conserved intracellular signaling network that enables cells to adapt to variable environmental conditions (defects in protein homeostasis, nutrient deprivation, viral infection, and oxidative stress) and maintain homeostasis. The ISR attempts to restore cellular balance by reprogramming gene expression. Proper termination of ISR signaling is essential, as it enables cells to reactivate protein synthesis and resume normal function. However, if the stress is irremediable, the ISR triggers apoptosis to eliminate the damaged cell [1].

**Figure 1:**
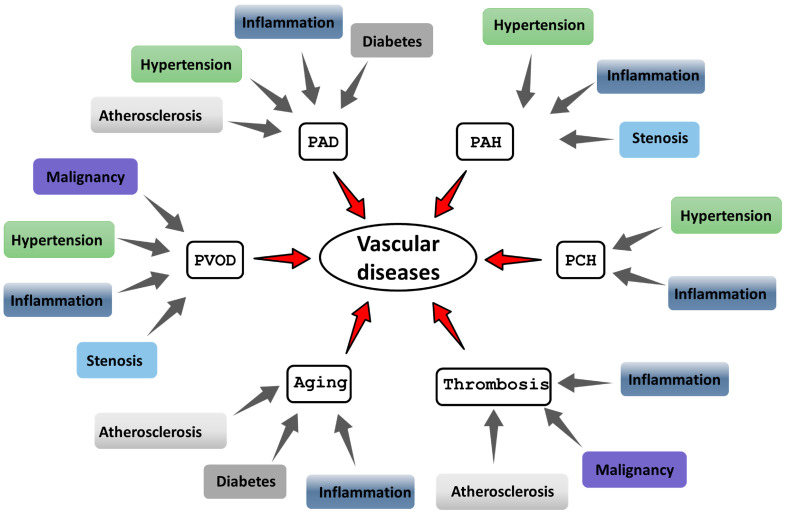
The diversity of diseases associated with vascular pathologies. Vascular diseases encompass a wide spectrum of pathological conditions, ranging from prevalent peripheral artery disease to rare cases of pulmonary veno-occlusive disease. Notwithstanding the role of genetic diseases, the predominant etiologies of the majority of vascular pathologies comprise inappropriate lifestyle behaviors (sedentary behavior, smoking, obesity), injuries, immune system disorders and infections, atherosclerosis, and hypertension. The blue and green boxes depict the vascular diseases and their causes, respectively, described in this review article.

As the cell layer lining the entire vascular system, the endothelium is uniquely exposed to physical injury, shear stress, and other damaging factors, even at normal blood pressure [2]. Since a key function of the endothelium is to supply organs and tissues with nutrients and oxygen, its healthy functioning is critical for maintaining physiological homeostasis. Therefore, diseases involving endothelial dysfunction can lead to serious complications and even death. Vascular diseases represent a major global health burden, significantly reducing quality of life and increasing mortality. A subset of cardiovascular diseases is linked to dysregulation of the ISR, involving either its overactivation or inadequate response. The ISR pathway is implicated in diseases like peripheral artery disease (PAD), pulmonary arterial hypertension (PAH), pulmonary capillary hemangiomatosis (PCH), pulmonary veno-occlusive disease (PVOD), thrombosis, and aging, which leads to a series of comorbidities (Figure 1).

The main external damaging factors are inflammation, hypertension, diabetes, and atherosclerosis (Figure 1).

Chronic inflammation plays a central role in the development and progression of many vascular diseases [3,4,5]. Among them are various forms of pulmonary hypertension, including PAH, PCH, PVOD, and chronic venous insufficiency. Inflammatory processes contribute to endothelial dysfunction and apoptosis, plaque instability, and ultimately, the narrowing and blockage of arteries occurring in atherosclerosis and related conditions like PAD.

PAD affects approximately 17% of the global population, with prevalence increasing with age [6] and strongly associated with increased cardiovascular morbidity and mortality, including heightened risks of myocardial infarction, cerebrovascular events, and major adverse coronary events [7,8]. Therapy for PAD includes the reduction in low-density lipoprotein (LDL) levels along with antithrombotic agents, anti-inflammatory therapies, vascular regenerative approaches, and cardiometabolic drugs. PAD is directly linked to the atherosclerosis [9].

Atherosclerotic cardiovascular diseases are the leading causes of morbidity and mortality worldwide, accounting for 50% of all deaths in Western societies [10]. Risk factors include smoking, obesity, physical inactivity, hyperlipidemia, diabetes mellitus, autoimmune diseases, and high levels of Lipoprotein(a) (Lp(a)) [10,11]. Atherosclerosis is considered a chronic inflammatory disease, the main causes of which are endothelial dysfunction, due to chronic inflammation or altered shear stress, and oxidized LDLs [12]. Progressive atherosclerotic plaques can narrow the arterial lumen, reducing blood flow and inducing ischemia [13]. However, even plaques that do not critically limit blood flow can become unstable, leading to thrombotic occlusion and sudden ischemia [10].

Thrombosis refers to the formation of clots within blood vessels, which reduce or block blood flow and pose serious health risks, including pulmonary embolism and stroke [14]. Contributing factors include blood vessel injury (including due to chronic illnesses), altered blood flow, genetic predispositions, elevated estrogen levels (estrogen-containing contraceptives, hormone replacement therapy, pregnancy), and prolonged restricted movement [15,16]. Compression therapy and direct oral anticoagulants are commonly used to reduce the risk of complications [17,18].

PAH is a rare disease affecting approximately 6 people per million per year. PAH is characterized by structural alterations in pulmonary arteries and progressive vascular occlusion, leading to increased right ventricular afterload, right heart failure, and death [19]. PAH may be inherited or it may be caused by connective tissue diseases, human immunodeficiency virus infection, portal hypertension, congenital heart disease, and schistosomiasis [19]. Current treatment strategies focus on preventing right heart decompensation, alleviating symptoms, and targeting pulmonary arterial endothelial dysfunction.

Pulmonary capillary hemangiomatosis (PCH) is a rare and aggressive form of pulmonary hypertension characterized by abnormal capillary proliferation within the alveolar interstitium. The etiology of PCH is not clear. Initially, it was considered to be a low-grade vascular neoplasm. More recent studies suggest that PCH may arise as a primary disorder due to EIF2AK4 mutations or secondarily in response to chronic venous hypertension (e.g., left heart disease) or toxic exposure (e.g., chemotherapeutics). Notably, therapies targeting PAH are contraindicated due to the risk of life-threatening pulmonary edema, leaving lung transplantation as the only definitive treatment.

Pulmonary veno-occlusive disease (PVOD) is a rare occlusive disorder (1 to 2 cases per 10 million) of the pulmonary veins associated with various risk factors, including certain medications, bone marrow transplantation, viral infections, endothelial injury, toxins, ionizing radiation, autoimmune diseases, pregnancy, malignancy, and heritable mutations [20]. The etiology of familial PVOD is analogous to that of PCH, and it is caused by mutations in the EIF2AK4 gene. PVOD is considered a rare and rapidly progressive subtype of PAH, with most patients dying within two years of diagnosis and is characterized by progressive obstruction of small pulmonary veins, leading to elevated pulmonary arterial pressure, right-sided heart failure, and pulmonary fibrosis. Bilateral lung transplantation remains the only curative option, as standard pulmonary vasodilators are largely ineffective in PVOD.

Although there is currently no cure for most vascular diseases, existing treatment strategies focus mainly on managing risk factors, slowing disease progression, and alleviating symptoms. Therapeutic approaches range from lifestyle modifications and pharmacological interventions to surgical correction. The specific treatment approach depends on the type and severity of the vascular disease and may include medications aimed at reducing inflammation, alleviating pain, and modulating immune system activity.

However, these treatments are largely symptomatic and do not address the underlying molecular causes of disease. This highlights the urgent need for the development of novel targeted therapies. Achieving this requires a more detailed understanding of the molecular pathways involved in the onset and progression of vascular diseases, particularly those signaling networks that remain under-explored in this context. Furthermore, a comprehensive analysis is required to elucidate the impact of these signaling pathways modulation on the progression of vascular diseases and clinical outcomes.

One such pathway is the integrated stress response (ISR)—a fundamental cellular mechanism that maintains proteostasis and orchestrates adaptive responses to stress. Despite its well-characterized roles in other organ systems, the ISR has received relatively little attention in the field of vascular biology. Emerging evidence, however, highlights that ISR signaling contributes to key pathological processes such as endothelial dysfunction, inflammation, apoptosis, atherosclerosis, and PVOD. ISR signaling represents a critical node in vascular diseases. This concept is supported by convergent evidence from transcriptomic, proteomic, and functional studies. In human symptomatic carotid plaques, expression of ISR effectors such as CHOP (DDIT3) is significantly elevated, consistent with widespread ER stress and apoptosis across vascular cell types [21]. In murine models, unfolded protein response activation is detectable at all stages of atherosclerotic plaque development [22], and genetic deletion of CHOP reduces necrotic core size and plaque instability [23]. ISR activation via lipid overload further exacerbates atherosclerosis, while its pharmacological inhibition diminishes lesion burden and inflammatory gene expression [24].

Recent proteomic analyses using tandem mass tag labeling (TMT–LC-MS) demonstrated that cardiac ischemia/reperfusion (I/R) induces global suppression of protein translation through the PERK–eIF2α axis, thereby limiting mitochondrial oxidative stress and cell death [25].

Importantly, recent single-cell RNA sequencing of arteries from a murine model of Hutchinson–Gilford progeria syndrome revealed that ISR-related transcriptional activity originates predominantly from non-endothelial vascular cells—especially smooth muscle cells—underscoring cell type-specific stress responses within the vasculature [26].

Furthermore, single-nucleus RNA sequencing (snRNA-seq) of aortic tissue from ApoE-/- mice expressing progerin specifically in VSMCs revealed selective upregulation of ER stress and ISR-related genes in VSMCs, including ATF4, CHOP (DDIT3), and HSPA5 (BiP/Grp78). This activation of the maladaptive ISR was shown to promote VSMC apoptosis and accelerate atherosclerosis. Importantly, pharmacological inhibition of PERK mitigated these effects by downregulating ISR gene expression, preserving VSMC viability, and reducing plaque progression [27]. Together, these multi-omics and mechanistic findings substantiate the role of the ISR as a functional and cell-specific regulator of vascular disease progression.

This review examines the current understanding of ISR signaling in vascular pathophysiology, with a particular focus on the roles of individual ISR kinases and their pharmacological modulators. In addition, it introduces a critical conceptual framework: the ISR functions as a double-edged sword, with outcomes—protective or deleterious—determined by the cellular context, the nature and duration of stress, and the specific kinase activated. We argue that successful therapeutic targeting will require a shift away from broad ISR inhibition or activation toward precise, context-dependent modulation.

Figure 1 shows the diversity of diseases associated with vascular pathologies. Vascular diseases encompass a wide spectrum of pathological conditions, ranging from peripheral artery disease (PAD), pulmonary arterial hypertension (PAH), pulmonary capillary hemangiomatosis (PCH), pulmonary veno-occlusive disease (PVOD), thrombosis, and aging. All the used abbreviations throughout the text are listed in the Supplementary Material.

## 2. The Molecular Mechanisms of the Integrated Stress Response (ISR)

The integrated stress response (ISR) is an evolutionarily conserved eukaryotic signaling network that coordinates translational control in response to diverse stress signals. At the core of this system is the phosphorylation of the α-subunit of eukaryotic translation initiation factor 2 (eIF2α) at serine 51 by various eIF2α kinases, which reduces global protein synthesis while enabling selective translation of stress-responsive transcripts, most notably ATF4 in vertebrates. The main event of the ISR is the phosphorylation of eIF2α as a central regulator of translation, emerging early in eukaryotic evolution as a universal mechanism for adapting to nutrient deprivation and other fundamental stressors. While this core mechanism is widely conserved, the upstream activating kinases and downstream effectors involved in transcriptional response exhibit considerable evolutionary plasticity [28].

**Figure 2:**
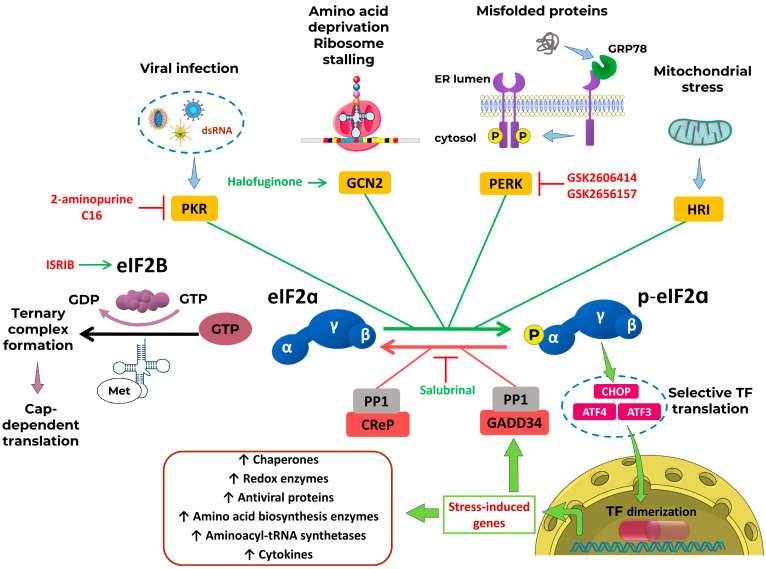
The mechanisms of the integrated stress response (ISR) pathway and its pharmacological modulation. The ISR is initiated by stress-sensing kinases (PERK, GCN2, PKR, HRI), which phosphorylate eIF2α. Upon ISR activation, global cap-dependent translation (via impaired ternary complex formation) is suppressed while enabling selective translation of ISR transcription factors (e.g., ATF4/ATF3) and induction of stress-adaptive gene programs. Negative feedback is provided by GADD34/CReP-containing phosphatase complexes. Pharmacologic modulators are indicated: GSK2606414/GSK2656157 (PERK inhibitors), 2-aminopurine/C16 (PKR inhibitors), halofuginone (GCN2 activator), and salubrinal (inhibitor of eIF2α dephosphorylation by PP1–PPP1R15 complexes, prolonging p-eIF2α signaling). Inhibitors are shown in red, while activators are in green.

The main participants and regulators of this pathway are depicted in Figure 2.

Among eIF2α kinases, GCN2 (General Control Non-Derepressible 2, EIF2AK4) is the most widely conserved. It is the only eIF2α kinase found across all eukaryotic kingdoms: fungi (e.g., *S. cerevisiae*, *N. crassa*), plants, animals, and many protists [28,29]. GCN2 primarily responds to amino acid starvation and ribosomal stress. In *S. cerevisiae*, it is the sole eIF2α kinase present; consequently, the ISR in yeast is traditionally referred to as the General Amino Acid Control pathway. In filamentous fungi, such as *Neurospora* and *Aspergillus*, the same pathway is also known as Cross-Pathway Control [30].

The HRI (heme-regulated inhibitor, EIF2AK1), like GCN2, also appears to have emerged early in eukaryotic evolution. It is found in fungi such as *S. pombe* and in some protists, but its orthologs are notably absent in widely used invertebrate models: *C. elegans* and *D. melanogaster* [29]. The HRI is activated in response to heme depletion and more recently has been recognized as a mediator of mitochondrial stress responses [31]. In vertebrates, the HRI acquired a specialized role in erythroid cells, where it regulates globin synthesis in response to heme availability during erythropoiesis [32].

PERK (Protein kinase RNA-like Endoplasmic Reticulum Kinase, EIF2AK3) functions as a sensor of endoplasmic reticulum stress. It is conserved across some protists, invertebrates, and vertebrates, but is absent in plants and fungi [29].

PKR (Protein Kinase R, EIF2AK2) is a cytosolic sensor of double-stranded RNA, with a pivotal role in innate immunity. PKR is restricted to vertebrates and is considered a relatively recent evolutionary innovation linked to antiviral innate immunity [28,29].

Interestingly, a related kinase known as PKZ, which contains Z-DNA/RNA-binding domains instead of double-stranded RNA-binding motifs present in PKR, is found exclusively in teleost fish, further highlighting lineage-specific expansions of ISR components [33].

Despite the divergence in regulatory domains, all four canonical eIF2α kinases share conserved catalytic domains [31] and utilize a common activation mechanism involving dimerization and autophosphorylation [34]. These kinases have overlapping functions and can act cooperatively to fine-tune cellular responses to stress; for example, all of these eIF2α kinases become subject to oxidative stress conditions [31].

Recent studies also identified non-canonical kinases that modulate ISR activity. MARK2 was shown to directly phosphorylate eIF2α under proteotoxic stress, promoting translational attenuation and stress adaptation [35]. Additionally, FAM69C has recently been characterized as a serine-threonine kinase required for eIF2α phosphorylation and ATF4 induction in microglia, linking it functionally to canonical ISR signaling [36].

Effector responses downstream of eIF2α phosphorylation also exhibit significant evolutionary plasticity. In *S. cerevisiae*, the key ISR effector is the transcription factor Gcn4p, which induces the expression of genes involved in amino acid biosynthesis, aminoacyl-tRNA synthetases, and general stress response pathways [30]. In *C. elegans*, the main ISR effector is ATFS-1, a transcription factor primarily involved in the mitochondrial unfolded protein response [37]. In *D. melanogaster*, the Crc (cryptocephal) protein, an ortholog of mammalian ATF4 [38] and recently described Xrp1 [39], performs a functionally similar role in mediating translational stress responses.

In vertebrates, ATF4 is the prototypical ISR effector, regulating a broad transcriptional program upon eIF2α phosphorylation [40]. Despite their divergent evolutionary origins and regulatory mechanisms, these transcription factors converge functionally by activating overlapping sets of genes, including those involved in antioxidant defense, molecular chaperoning, amino acid transport, and cellular metabolism [41].

Having outlined the evolutionary origins and general architecture of the ISR, we now turn to a detailed description of its canonical mechanism in vertebrates.

Translation initiation begins with the formation of the ternary complex (TC), comprising the eIF2, which consists of α, β, and γ subunits, GTP, and the initiator methionyl-tRNA (Met-tRNAiMet). This is followed by binding TC with the 40S ribosomal subunit bound to eIF1, eIF1A, eIF3, and eIF5, resulting in assembly of the 43S pre-initiation complex [42].

The 43S complex then binds to the capped 5′ end of the mRNA, facilitated by the eIF4 factors (A, B, E, G) and the poly(A)-binding protein, forming the 48S initiation complex. This complex scans the mRNA leader region for an AUG start codon [30]. Upon base-pairing of the Met-tRNAi^Met^ anticodon with AUG, eIF5 stimulates GTP hydrolysis on eIF2 and eIF1 dissociates. This leads to the release of eIF2-GDP and promotes joining of the 60S ribosomal subunit, a process facilitated by a second GTPase, eIF5B, to form the translation-competent 80S initiation complex.

The eIF2-GDP released in this process must be recycled into eIF2-GTP by the guanine nucleotide exchange factor eIF2B. eIF5 stabilizes the GDP-bound form of eIF2, acting as a GDP dissociation inhibitor, and eIF2B effectively displaces eIF5 and catalyzes GDP-to-GTP exchange [43]. This exchange is inhibited by canonical or non-canonical eIF2α kinases that phosphorylate Ser51 on the α-subunit of eIF2.

Phosphorylated eIF2α (p-eIF2α) acts as a competitive inhibitor of eIF2B. Because eIF2 is more abundant than eIF2B, even small amounts of phosphorylated eIF2α are sufficient to sequester much of the available eIF2B, leading to global attenuation of 5′-cap-dependent translation [44].

Despite this suppression of general translation, specific transcripts containing upstream open reading frames in their 5′ untranslated regions such as ATF4, ATF5, CHOP, and GADD34 are preferentially translated [31]. This selective translation may occur via re-initiation mechanisms or through cap-independent internal ribosome entry site-mediated recruitment.

Elevated ATF4 levels facilitate dimerization with other basic leucine zipper (bZIP) DNA-binding proteins, such as C/EBP isoforms, leading to transcriptional activation of genes involved in amino acid synthesis and transport, mRNA turnover, redox regulation, and ISR feedback regulation [45]. ATF4 also exerts cytoprotective effects in various stress contexts [46].

Activation of the ISR promotes the selective translation of specific mRNAs encoding regulatory proteins. As a result, the magnitude and duration of ISR signaling must be tightly regulated to prevent inappropriate or prolonged translational repression. This regulation is achieved through a negative feedback loop involving protein phosphatase 1 (PP1) complexes, which consist of the catalytic subunit PP1c and one of two regulatory subunits: GADD34 (*PPP1R15A*), which is induced during ISR, or the constitutively expressed paralog CReP (*PPP1R15B*). CReP maintains low basal levels of eIF2α phosphorylation under non-stress conditions, thereby sustaining normal translation [47]. During stress, GADD34 is transcriptionally induced by ATF4 and recruits PP1c to dephosphorylate eIF2α, restoring protein synthesis once homeostasis is re-established and promoting cell survival. However, the ISR is often described as a double-edged sword: while it can facilitate recovery and adaptation, unresolved or chronic stress can shift ISR output toward pro-apoptotic signaling. In such cases, continued ATF4 activity may promote the expression of death-inducing proteins, including CHOP, thereby contributing to cell death rather than survival [31].

While the classical ISR pathway centers on eIF2α phosphorylation, emerging evidence highlights additional layers of regulation involving alternative translation initiation factors. Recent studies have revealed additional initiation factors that contribute to non-canonical ISR regulation. eIF2D and DENR/MCT-1, which can deliver aminoacyl-tRNAs to the ribosome independently of eIF2, are essential for ATF4 translation in *Drosophila* and humans [48,49,50]. Moreover, eIF3d, an alternative cap-binding protein, maintains ISR signaling under chronic stress by promoting GCN2 translation and indirectly regulating ATF4 via modulation of m6A demethylation [51]. These findings uncover auxiliary mechanisms of translation control integrated into the ISR.

The ISR has drawn significant biomedical interest partly due to its association with many diseases including cardiovascular and ischemic conditions, reflecting the ISR’s critical role in cellular homeostasis. It also participates in normal physiological processes. For instance, the vascular endothelium is constantly exposed to mechanical stress, cyclic shear forces [52], and fluctuating nutrient and oxygen levels. Long-term stress conditions could lead to endothelial dysfunction. This includes changes in the functional phenotype, hemostasis, local vascular tension, and redox balance. Changes in endothelial cell phenotype include diminished production of nitric oxide (NO) and anti-coagulant properties [53]. Endothelial permeability is also impaired at least in part through a vinculin-dependent mechanism [54]. Upon injury or hypoxia, endothelial cells activate the ISR to preserve integrity and metabolic balance [55]. Recent studies suggest that ISR components, including ATF4 and PERK, are essential for maintaining endothelial function under such stress conditions [56].

At the moment, different ISR modulatory compounds have been developed. In this review, we describe the role of the ISR pathway in vascular homeostasis as well as the possible application of ISR modulators in vascular diseases. The pharmacological properties of the discussed modulators and their applications are depicted in Table 1.

## 3. ISR Signaling and Its Modulators in Vascular Normal and Pathological Physiology

### 3.1. PERK

The PERK protein kinase is encoded by the EIF2AK3 gene and constitutes one of the major ER stress signaling pathways, along with the ATF6 and IRE1 cascades. The accumulation of misfolded proteins within the cell results in the dissociation of the ER-resident chaperone GRP78 from PERK. This process subsequently leads to the formation of a PERK homodimer, which is then activated via autophosphorylation. PERK kinase activity has been demonstrated to mediate not only the activation of ISR signaling, but also to stimulate antioxidant defense by promoting the dissociation of the NRF2 molecule from its complex with the KEAP1 protein. In addition, PERK kinase activity regulates Ca^2+^-dependent apoptosis by directly interacting with calcineurin, a component of the FKBP12.6 signaling pathway [57].

Mutations in the EIF2AK3 gene underscore the indispensable nature of PERK for organismal homeostasis: biallelic loss-of-function mutations cause Wolcott–Rallison syndrome, an autosomal–recessive disorder marked by neonatal or early-onset diabetes, epiphyseo-metaphyseal dysplasia, fulminant hepatic failure, and high childhood mortality [58].

PERK signaling plays a role in the physiology of diverse tissues and its activation is also important in the endothelium, where it drives endothelial-to-mesenchymal transition (EndoMT) and fine-tunes mitochondrial fatty-acid β-oxidation and tricarboxylic-acid-cycle flux—metabolic and phenotypic changes essential for endocardial cushion remodeling during aortic valve morphogenesis [59].

PERK is also essential for physiological angiogenesis: VEGFA engagement of VEGFR2 activates PERK via a PLCγ/mTORC1 cascade, and the ensuing PERK-dependent phosphorylation of eIF2α coordinates endothelial growth in Matrigel^TM^ plug assay [60]. Consistent with this role, hypoxia/re-oxygenation elevates PERK activity and its transcriptional effector ATF4; the PERK–ATF4 axis upregulates the pro-angiogenic genes HIF-1α and VEGFA, reinforcing endothelial adaptation to oxygen stress. Pharmacological inhibition of PERK with GSK2606414 suppresses this induction, providing direct evidence that PERK signaling is required for the upregulation of HIF-1α and VEGFA [61].

Conversely, in diabetic cardiomyopathy, VEGFB is upregulated, a cytokine that exacerbates cardiac dysfunction while concurrently suppressing the adaptive PERK–p-eIF2α axis. Melatonin alleviates cardiac dysfunction and increases PERK phosphorylation, whereas administration of another selective PERK inhibitor GSK2656157 abolishes this melatonin-mediated protection. Collectively, these findings highlight PERK kinase as an integral part of normal physiology and is essential for angiogenesis processes [62].

However, it should be noted that the pro-angiogenic activity of the PERK–p-eIF2α pathway is not invariably beneficial and can also drive pathological neovascularization. Accumulating evidence shows that sustained PERK activity facilitates tumor growth in multiple ways. For instance, under prolonged serum deprivation, cancer-associated fibroblasts (CAFs) from pancreatic ductal adenocarcinoma activate a PERK–p-eIF2α–ERK1/2 signaling cascade that is essential for their EndoMT and the ensuing tumor neo-angiogenesis. This transition endows CAFs with endothelial-like properties in vitro and enables their direct incorporation into the tumor vasculature in vivo. Pharmacological inhibition of PERK with GSK2606414 markedly reduces micro-vessel density and suppresses tumor growth, whereas pharmacological activation with CCT020312 has the opposite effect, amplifying EndoMT, angiogenesis, and tumor progression [63].

Furthermore, PERK signaling amplifies the activity of peptidyl-glycine α-amidating mono-oxygenase (PAM)—the sole enzyme that converts C-terminal glycine-extended peptides into bioactive α-amidated hormones such as the strongly pro-angiogenic adrenomedullin. By enlarging the repertoire of secreted, angiogenic peptides, PERK-PAM coupling deepens the vascular-promoting phenotype of stressed tumors [64,65]. In support of the hypothesis that PAM activation in tumors is PERK-dependent, it was demonstrated that the use of GSK2606414 suppressed PAM activity, whereas the PERK agonist CCT020312 intensified PAM cleavage, triggering cytosolic signaling of the fragment that fosters tumor migration and angiogenesis [64]. Consistently, another PERK inhibitor, GSK2656157, also exhibits robust growth inhibition and anti-angiogenic activity across multiple human xenograft tumors in mice [66].

Collectively, these observations underscore the pivotal role of PERK kinase-driven integrated stress response signaling in promoting angiogenesis. Although angiogenesis is normally a beneficial, tightly regulated process, during tumorigenesis, the same pathway becomes a key driver of pathological neo-angiogenesis within tumors [67,68]. Importantly, PERK’s pathological vascular influence is not confined to cancer: accumulating evidence shows that this stress response pathway likewise orchestrates other forms of aberrant angiogenesis and maladaptive vascular remodeling, contributing to atherosclerosis, restenosis, and thrombosis—issues that are described in detail below.

#### 3.1.1. PERK Kinase as a Therapeutic Target in Atherosclerosis

Recent evidence implicates PERK-driven integrated stress response signaling in the pathogenesis of atherosclerosis. Specifically, the gut microbiota metabolite trimethylamine-N-oxide (TMAO) activates the PERK/p-eIF2α/ATF4/ATF3 cascade in vascular endothelial cells, eliciting oxidative stress, EndoMT, and apoptosis. In ApoE-/- mice, these TMAO-induced events aggravate plaque formation and hemodynamic dysfunction, whereas pharmacological inhibition of PERK with GSK2606414 markedly attenuates disease progression [69]. In addition, it was shown that TMAO can bind to and directly activate PERK, provoking mitochondrial stress and the generation of mitochondrial reactive oxygen species in endothelial cells, thereby amplifying inflammatory signaling [70]. Collectively, these findings indicate that pharmacological inhibition of PERK signaling may represent a promising strategy to prevent TMAO-driven atherogenesis.

Beyond TMAO, oxidized low-density lipoprotein (ox-LDL) constitutes another major pro-atherogenic insult: ox-LDL accumulates in the arterial wall, provokes endothelial oxidative stress and apoptosis, and thereby accelerates plaque formation [71]. Analogous to TMAO, ox-LDL provokes endothelial dysfunction through activation of the PERK/p-eIF2α signaling pathway. PERK suppression by siRNA attenuated the negative effects of ox-LDL and restored endothelial NO synthase activity [72].

Ox-LDL activates PERK signaling through several upstream routes: by upregulating CNPY2, LOX1, and NOX4 and activating the non-canonical cGAS-STING pathway. Each of these mechanisms is discussed in detail below.

##### CNPY2

Huang et al. demonstrated that ox-LDL activates the PERK/p-eIF2α signaling pathway by upregulating canopy FGF-signaling regulator 2 (CNPY2). CNPY2 not only functions as an ER co-chaperone that enhances PERK activity but is also a secreted HIF-1α-responsive angiogenic factor. CNPY2 aggravated the process of atherosclerosis in ApoE-/- mice in vivo and induced endothelial cell injury in vitro; the latter was markedly attenuated when the cells were treated with the PERK inhibitor GSK2606414 [73]. Recently, CNPY2 has also been implicated in the pathogenesis of angiotensin II (Ang II)-induced hypertension. Endothelial apoptosis—a key element of hypertensive vascular injury—is mediated, at least partly, through the CNPY2/PERK/Ca^2+^/CaMKII/Drp1 and CNPY2/PERK/p-eIF2α/CHOP cascades triggered by Ang II. The antihypertensive peptide angiotensin-(1–9) dampens both cascades and thereby reduces endothelial cell death. Supporting the central role of PERK, the selective inhibitor GSK2606414 markedly reduces Ang II-evoked apoptosis, whereas the PERK activator CCT020312 inhibits the protective effect of angiotensin-(1–9) [74]. Collectively, these findings indicate that the CNPY2 protein functions as an additional component of PERK signaling, suggesting its potential as a novel and promising therapeutic target for vascular disorders, including atherosclerosis.

##### LOX-1/NOX4

In addition to CNPY2 ox-LDL induces expression of lectin-like oxidized-LDL receptor-1 (LOX-1), the principal endothelial scavenger for modified lipoprotein, which is required for the ox-LDL-driven upregulation of the ER-resident NADPH oxidase NOX4. Downregulation of either LOX-1 or NOX4 suppresses PERK/p-eIF2α/CHOP signaling and prevents ox-LDL-triggered endothelial apoptosis. Notably, LOX-1 expression is also elevated by other pro-atherogenic stimuli, such as hypertension, diabetes, hyperglycemia, and disturbed shear stress, suggesting that this pathway may operate across a wide spectrum of vascular pathologies, well beyond ox-LDL exposure [75,76].

NOX4-driven oxidative stress provides the link between hyperhomocysteinaemia and PERK activation. Hyperhomocysteinemia plays a vital role in the development of atherosclerosis and has been associated with the activation of the PERK/p-eIF2α/CHOP signaling pathway, increased NOX4 expression, and increased cell apoptosis. The suppression of PERK signaling with catapol has been shown to significantly alleviate the negative effects of homocysteine on endothelial cells and to reduce NOX4 expression—the detrimental factor in these conditions [77].

Conversely, several studies suggest that NOX4 may exert a protective effect in endothelial cells by downregulating the expression of soluble epoxide hydrolase-2, a pro-inflammatory, pro-atherogenic enzyme. Accordingly, NOX4 deficiency leads to upregulation of ER stress markers, including BiP (Grp78), IRE1α, p-PERK, and ATF6 [78]. In this context, the balance of NOX4 activity must be considered: both excessive activation and loss of NOX4 are detrimental, necessitating a more thorough approach to the development of NOX4 suppression strategies in the context of ox-LDL-induced atherosclerosis.

##### (cGAS)-STING Pathway

Treatment of human coronary artery endothelial cells with ox-LDL increases cytosolic double-stranded DNA (dsDNA), predominantly of mitochondrial origin. This increases cGAMP generation and promotes the phosphorylation and physical association of STING and PERK, thereby activating the non-canonical STING-PERK signaling pathway. Similarly, significant increases in cytoplasmic dsDNA were also observed in human atherosclerotic plaques and in the vascular wall of ApoE-/- mice on a high-fat diet. This was accompanied by increased phosphorylation of STING and PERK, indicating the engagement of the same non-canonical pathway. This highlights new therapeutic approaches for improving vascular endothelial dysfunction and atherosclerosis [79].

It is worth noting that ox-LDL upregulates the pro-apoptotic factor CHOP through PERK-dependent eIF2α phosphorylation, engaging a principal arm of ISR signaling [71,80]. The same PERK/p-eIF2α/CHOP cascade is activated by tunicamycin, a well-established inducer of ER stress [81]. Collectively, these findings implicate the ISR, particularly the PERK axis, as a central driver of endothelial apoptosis, a defining feature of atherosclerosis.

#### 3.1.2. Role of PERK Kinase Signaling in Restenosis and Thrombosis

Beyond atherosclerosis, PERK/p-eIF2α signaling has also been implicated in restenosis and thrombosis. In preclinical models, administration of the selective PERK inhibitor GSK2606414 mitigated these vascular abnormalities—markedly reducing the intima-to-media ratio, preserving luminal area, maintaining endothelial proliferative capacity, and lowering expression of the thrombogenic coagulation factor III/CD142 (tissue factor) [82]. GSK2606414 has been demonstrated to enhance post-angioplasty re-endothelialization and suppress the pro-inflammatory microenvironment associated with restenosis, leading to a reduction in inflammatory factors such as IL1-β, IL6, and VCAM1 [83]. The administration of GSK2606414 in adverse post-angioplasty events has proven effective, so special delivery tools for GSK2606414 in in vivo applications have been developed [82]. These observations suggest the clinical potential of PERK inhibitors not only in atherosclerosis, but also in a number of other vascular pathologies.

Taken together, the evidence reviewed in this section firmly establishes the PERK/p-eIF2α arm of ISR signaling as an essential regulator of angiogenesis in both a healthy state and disease. Under physiological conditions, this pathway regulates vessel growth and maintains tissue homeostasis. However, under pathological stress, it becomes deleterious—enhancing tumor neovascularization, atherosclerotic plaque expansion, and promoting endothelial apoptosis. Thus, it is evident that selective PERK inhibitors, such as GSK2606414 and GSK2656157, hold considerable potential as a novel therapeutic strategy for vascular diseases.

Intriguingly, the ISR enhancer salubrinal, which prolongs eIF2α phosphorylation and would be expected to amplify PERK-mediated stress, has instead been shown to attenuate atherosclerotic lesion formation and reduce endothelial apoptosis [71,75,84]. This apparent paradox probably reflects a context-dependent shift in ISR output away from the pro-apoptotic transcription factor CHOP toward the pro-survival mediator ATF4. Nonetheless, further comprehensive investigation is required to define the precise molecular mechanisms by which salubrinal confers vascular protection and to determine its optimal therapeutic window.

These findings underscore the central idea proposed in this review: the ISR is not inherently beneficial or harmful, but rather functions as a context-dependent regulatory system. Its cellular outcome—adaptive or pathological—depends on the nature, duration, and intensity of stress, as well as on downstream signaling bias (e.g., CHOP vs. ATF4 dominance). This duality explains why both ISR inhibitors and enhancers may yield therapeutic benefit under distinct conditions. Understanding and manipulating this balance—rather than simply blocking or activating the ISR—will be essential for developing safe and effective vascular therapies.

### 3.2. GCN2

Protein kinase GCN2 (EIF2AK4) is known to undergo activation in response to various stress stimuli, including amino acid starvation, UV irradiation, metabolic perturbations, and oxidative stress conditions. GCN2 has been shown to function as a sensor of uncharged tRNA accumulation, ribosome stalling, and collisions [85]. It also plays an important role in a number of physiological processes including memory formation, regulation of cellular metabolism, and immune and vascular physiology [86].

Evidence from EIF2AK4 loss-of-function mutations in PVOD/PCH suggests that GCN2 plays a crucial role in maintaining pulmonary vascular homeostasis, although the exact functional outcome of GCN2 activation is likely to be highly context-dependent.

This assertion is supported by the fact that biallelic mutations in the *EIF2AK4* gene cause pulmonary vascular dysfunction, which is classified as either pulmonary veno-occlusive disease (PVOD) or pulmonary capillary hemangiomatosis (PCH), depending on the predominant histopathological features observed.

PVOD is a rare autosomal recessive form of pulmonary hypertension (PAH) characterized by a progressive increase in pulmonary vascular resistance, resulting in right heart failure and high mortality [87,88]. Key clinical PVOD features include reduced diffusing lung capacity for carbon monoxide, precapillary PH, interlobular septal thickening, mediastinal lymphadenopathy, and centrilobular ground-glass opacities [87]. Molecular hallmarks of PVOD encompass the upregulation of heme oxygenase-1, pro-apoptotic CHOP expression, diminished ATF3 function, leading to sustained p38 MAPK phosphorylation, and increased collagen I deposition in the pulmonary vascular wall [89,90].

Pulmonary capillary hemangiomatosis (PCH) is characterized by a markedly atypical proliferation of capillaries. Alveolar capillaries invade the pulmonary interstitium, pulmonary veins and arteries, alveolar walls and septae, and other intrathoracic structures in a neoplastic manner. This leads to hypoxemia and dyspnea [91].

A common feature of PVOD and PCH is abnormal regulation of angiogenesis due to mutations in the GCN2 gene, underscoring the significance of this kinase in normal vascular physiology [87,92]. Currently, lung transplantation remains the only effective treatment for PVOD and PCH, despite pharmacological approaches proposed for symptom alleviation. This underscores the critical need for deeper research into the cellular and molecular mechanisms governing GCN2 signaling to develop effective therapies [87].

The known mechanism by which GCN2 regulates angiogenesis involves driving the endothelial expression of pro-angiogenic factors, such as VEGFA and cystathionine-γ-lyase, through the GCN2/p-eIF2α/ATF4 pathway [93]. Furthermore, tryptophanol, a competitive inhibitor of tryptophanyl-tRNA synthetase and thus an activator of GCN2, has been shown to have a protective effect on endothelial cells under high-glucose conditions by increasing GAPDH activity, reducing ROS formation, and generating advanced glycation end products—hexosamine and polyols [94].

Experimental knockdown of GCN2 in tumor cells has been shown to impair tumor progression and reduce angiogenesis in vivo, underscoring the dependence of tumor survival in nutrient-deprived microenvironments on GCN2–ATF4-mediated metabolic adaptation [95].

#### 3.2.1. Metabolic Roles of GCN2

GCN2 plays a pivotal role in cellular adaptation to nutritional and metabolic stress. Initially identified as a sensor of amino acid deprivation, GCN2 is now recognized as a central regulator of the ISR, coordinating homeostasis in response to a wide spectrum of stresses, including not only amino acid scarcity but also glucose deprivation, oxidative damage, UV irradiation, hypoxia, proteotoxic stress, and impaired ribosomal function [96,97,98,99,100]. A particularly novel finding is that GCN2 can be activated independently of uncharged tRNA accumulation. Ribosome stalling and collisions have been identified as alternative mechanisms of GCN2 activation. These processes are mediated through interaction with the ribosomal P-stalk complex (P1/P2/uL10), which can directly activate GCN2 kinase activity [101,102]—even in the absence of tRNA binding. This highlights GCN2’s role as a broader sensor of translational stress, linking ribosome dynamics to metabolic adaptation.

GCN2 activation leads to upregulation of ATF4, initiating a transcriptional program that coordinates multiple adaptive processes. Among these are increased expression of amino acid transporters [103,104,105,106], enhanced amino acid recycling, and induction of autophagy-related genes, including ATG5, ATG12, and LC3 [107]. These adaptations collectively improve amino acid efficiency and preserve proteostasis during stress conditions [108]. Importantly, the induction of autophagy by GCN2 occurs not only through ATF4 activation, but also indirectly via inhibition of mTORC1 signaling (e.g., through GCN2-mediated induction of Sestrin2 or phosphorylation of FBXO22 leading to mTOR ubiquitination), highlighting the intricate crosstalk between the ISR and major nutrient-sensing pathways [109]. This interplay between the ISR, AMPK, and mTOR pathways is crucial for metabolic regulation [110,111,112]. This posits GCN2 as a key metabolic integrator, fine-tuning the balance between anabolic growth and catabolic recycling in response to nutrient availability.

Beyond nutrient sensing, GCN2 influences key processes such as mitochondrial quality control and redox balance. Under nutrient-limited conditions, GCN2 enhances the expression of mitochondrial chaperones, such as TNF receptor-associated protein 1 (TRAP1), thereby reducing oxidative stress and supporting cell survival [113]. In cancer models, GCN2 activation has been shown to upregulate expression of xCT, BiP/Grp78, and other ATF4 target genes [114,115], mitigating oxidative stress.

GCN2 is also implicated in cell cycle regulation and genomic integrity. It promotes mitotic fidelity by phosphorylating and inhibiting Protein Phosphatase 1 (PP1), preventing premature dephosphorylation of mitotic regulators [116]. Loss of GCN2 leads to mitotic delays, chromosomal segregation errors, and heightened sensitivity to agents targeting mitotic progression, such as Aurora A inhibitors [116].

The GCN2-ATF4 axis also influences systemic physiology through endocrine factors. It has been shown to increase fibroblast growth factor 21 (FGF21) levels in skeletal muscle of mice under high-fat-diet conditions [117]. In pulmonary endothelial cells, inhibition of GCN2 exacerbates bleomycin-induced TGF-β1 expression and increases markers of mesenchymal transdifferentiation, including Twist family BHLH transcription factor (TWIST) [118]. Furthermore, GCN2 deficiency is associated with decreased expression of tight junction proteins ZO-1 and CD31, regulators of endothelial junctional integrity, suggesting a role in preserving endothelial barrier integrity [118]. At the transcriptional level, GCN2/ATF4 signaling can modulate the expression of endothelin-1 (ET-1), a potent vasoconstrictor involved in pulmonary hypertension. ET-1 is among the top upregulated genes following GCN2 activation [119]. While ET-1 contributes to vascular tone, its chronic overproduction is implicated in vascular inflammation, remodeling, and fibrosis, as seen in pulmonary hypertension [120]. In addition, inhibition of mTORC1, occurring in parallel with activation of GCN2 kinase, is a mechanism for the reduction in von Willebrand factor (vWF) expression [121]. Endothelial vWF has been shown to mediate angiotensin II-induced ET-1 expression through NOX-dependent superoxide production [122]. Thus, data supporting the regulation of ET-1 expression by GCN2 are available. Thus, the potential interaction of mTORC1 as a nutrient-sensing mechanism with ISR activation and ET-1-mediated vascular dysfunction highlights a potentially important therapeutic interplay between metabolism, oxidative stress, and endothelial signaling.

#### 3.2.2. Halofuginone

GCN2 kinase plays a pivotal role in vascular physiology by regulating both physiological and tumor angiogenesis. In conditions of amino acid starvation, GCN2 acts synergistically with ATF4 to drive tumor neo-angiogenesis by increasing the expression of the key pro-angiogenic factor VEGFA [95]. Consequently, the pharmacological inhibition of GCN2 is a promising strategy for suppressing pathological angiogenesis. Paradoxically, the GCN2 activator halofuginone, a synthetic halogenated derivative of febrifugine, also demonstrates therapeutic potential. Halofuginone inhibits glutamyl-prolyl-tRNA synthetase with high selectivity, thereby triggering GCN2-dependent ISR activation [123]. Halofuginone exerts endothelial-protective effects by suppressing the LPS-induced increase in pro-inflammatory mediators (TNF-α, IL-1β, IL-6, NOX-2, VCAM-1 and E-selectin) [124]. Other studies have also shown that halofuginone protects endothelial cells from the deleterious effects of hydrogen peroxide, thereby promoting the survival, proliferation, and pro-angiogenic abilities of vascular cells [125].

The effects of halofuginone extend beyond mere GCN2 activation. This compound significantly attenuates pulmonary vascular remodeling through regulation of K^+^/Ca^2+^ flux and PDGF/Akt/mTOR inhibition [126] and suppression of TGF-β1/Smad signaling [127]. This accounts for a significant and reversible inhibitory effect on alveolar hypoxia-induced pulmonary vascular remodeling and constriction, with a concomitant partial reversal of established PH [126]. In addition, halofuginone treatment has been shown to reduce inflammatory responses and right ventricular hypertrophy, while improving hemodynamic parameters and pulmonary vascular remodeling in the development of high-altitude pulmonary hypertension [127].

Therefore, the administration of halofuginone holds significant potential for the treatment of various vascular pathologies. Nevertheless, the existing literature has not yet adequately addressed the role of ISR signaling components in mediating the effects of halofuginone. In our estimation, when investigating the effects of halofuginone, it is imperative to incorporate an analysis of its impact on the ISR cascade as an integral component of experimental research. This approach is crucial for achieving a more profound comprehension of the mechanisms through which halofuginone exerts its effects and for elucidating its potential adverse effects. The necessity to explore the effects of halofuginone on the ISR signaling pathway is highlighted by evidence demonstrating that halofuginone reduced type 1 collagen production in injured arteries, thereby attenuating signs of intimal hyperplasia, a precursor of restenosis [128]. These findings are consistent with the results of a study on PVOD mechanisms, in which another ISR activator (salubrinal) was found to partially restore type 1 collagen synthesis, possibly due to increased ATF3 expression [90].

The profound implication of GCN2 kinase in cellular metabolism, in combination with the absence of effective treatments for diseases caused by GCN2 dysfunction, underscores the imperative for further exploration of the mechanisms underlying the function of this protein kinase. It can be posited that GCN2 modulators have the potential to establish a novel therapeutic paradigm for the treatment of various vascular diseases, analogous to the success of halofuginone in antitumor therapy, which is currently under active evaluation in clinical trials [129].

In summary, these findings exemplify the dual role of GCN2 signaling in vascular health and disease. On one hand, GCN2 activation promotes endothelial cell survival, upregulates VEGFA, and enhances stress resilience via the ATF4 pathway [93]. It also contributes to pathological neovascularization and vascular remodeling in both rare hereditary conditions such as PVOD and PCH—caused by EIF2AK4 loss-of-function mutations—and in tumor-associated angiogenesis [95]. On the other hand, the pharmacological GCN2 activator halofuginone has demonstrated therapeutic efficacy by suppressing tumor angiogenesis [130].

This apparent paradox highlights a broader and fundamental concept that recurs across ISR kinases: the functional outcome of ISR modulation is not fixed but profoundly context-dependent. It is shaped by variables such as cell type, stress intensity and duration, disease stage, and the relative dominance of downstream effectors like ATF4, CHOP, or ATF3. Therapeutic success will therefore require precise calibration rather than indiscriminate activation or suppression of ISR pathways. Achieving this will depend on in-depth mechanistic insights, biomarker-guided patient stratification, and tissue-targeted delivery strategies.

### 3.3. PKR

The protein kinase PKR is encoded by the EIF2AK2 gene and functions as a critical antiviral cellular defense mechanism, being activated by double-stranded RNA, a hallmark of viral RNA replication within the cell. Furthermore, PKR is known to be activated in response to a variety of other stimuli, including heat shock proteins, growth factors, ROS, cytokines, bacterial lipopolysaccharide, and ionizing radiation, among others. Nevertheless, PKR, by triggering ISR signaling, mainly suppresses the translation of viral proteins and ensures the synthesis of a number of antiviral factors [131]. PKR represents a unique ISR kinase that bridges classical stress signaling with broader inflammatory and senescence-related pathways. Unlike PERK and GCN2, PKR exerts strong immuno-modulatory functions through direct interaction with inflammasome components (NLRP1, NLRP3, AIM2) and regulates pro-inflammatory gene expression via JNK and STAT1 signaling [132]. In parallel, PKR activation has been implicated in promoting vascular and endothelial cell senescence, partly through suppression of SIRT1 and stabilization of p27^Kip1^ [133,134]. These dual roles position PKR as a central node at the intersection of inflammation, senescence, and stress adaptation.

#### 3.3.1. PKR as Regulator of Cellular Senescence and Proliferation

PKR is indispensable for normal vascular physiology, particularly within the endothelium. This is exemplified in peripheral artery disease, where endothelial expression of the angiogenic factor VEGFA is PKR-dependent [135]. Mechanistically, PKR upregulates VEGFA by activating the PI3K/Akt pathway in endothelial cells [136]. However, its role in pathology is context-dependent. For instance, PKR-mediated stimulation of angiogenesis is beneficial under hypoxic conditions [135], whereas in choroidal neovascularization, it contributes to pathological progression [136]. This duality underscores the necessity of developing PKR signaling modulators and evaluating their therapeutic efficacy across different disease contexts.

PKR also plays an important role in a number of other fundamental cellular processes, such as cellular senescence. As previously outlined, the ISR signaling mechanism is intricately linked to the pathophysiology of cellular senescence [137]. In particular, palmitate-induced senescence activates PKR signaling, which in turn leads to the suppression of the anti-senescent factor Sirtuin 1 through the activation of the JNK cascade [133]. Notably, the implementation of the PKR inhibitor 2-aminopurine or the JNK inhibitor SP600125 has been observed to attenuate endothelial cell senescence and partially restore SIRT1 activity [133]. It has also been reported that with age, there is an increase in PKR kinase activation in endothelial cells. This increase is responsible for Gasdermin D-mediated endothelial hyperactivation, which is characterized, among other features, by increased secretion of pro-inflammatory factors. These factors, in turn, contribute to the transformation of vascular smooth muscle cells into a secretory pro-senescent phenotype [138]. The implementation of siPKR in aged organisms exhibited a mitigating effect on vascular cell senescence, as evidenced by the suppression of senescence markers at the molecular level and the partial restoration of vascular morphology at the tissue level [138]. Accordingly, cellular senescence and organismal aging have been demonstrated to be associated with impaired PKR kinase function in vascular cells. These findings suggest that PKR modulators may hold significant potential as novel senotherapeutic agents.

PKR kinase has also been demonstrated to regulate the cell cycle. In an active state, this kinase suppresses the degradation of the cell cycle inhibitor protein p27^Kip1^, thereby preventing the G1- to S-phase transition of vascular smooth muscle cells [134,139]. Subsequent research determined that the antiproliferative effect of PKR is attributable to two factors: the stabilization of the p27^Kip1^ protein and the activation and phosphorylation of the Signal Transducer and Activator of Transcription-1 protein, an additional antiproliferative factor [140]. Furthermore, the antiproliferative properties of heparin have been demonstrated to impede the proliferation of vascular smooth muscle cells, thereby enhancing endothelial repair following injury and reducing the development of atherosclerosis. This process has been found to be dependent on the activation of PKR-mediated cell cycle arrest, a consequence of heparin’s direct binding to PKR and its subsequent activation [141]. However, PKR-dependent activation of STAT1 has been observed to elicit not only the arrest of vascular smooth muscle cell proliferation, but also the initiation of a pro-inflammatory cascade in macrophages, resulting in their subsequent polarization into the M1 phenotype. This, in turn, has been shown to promote the development of atherosclerosis [132]. These findings underscore the need to localize the application of specific PKR modulators to circumvent deleterious side effects.

#### 3.3.2. Role of PKR in Vascular Disorders

PKR plays an indispensable role in fundamental cellular processes, making it an essential component of the physiology of a wide range of pathological processes. Consequently, PKR abnormalities exacerbate hypertension induced by NG-Nitro-L-arginine Methyl Ester Hydrochloride, leading to an inflammatory response, cardiac/arterial remodeling, increased heart weight, upregulation of angiotensin expression, and expansion of the fibrosis zone. Notably, the use of the PKR inhibitor, compound C16, has been shown to reverse these deleterious effects [142]. A similar pattern is observed in vascular smooth muscle cells affected by fatty degeneration, where PKR contributes to the development of inflammatory reactions, ROS generation, calcification, and cell transformation, while the treatment of these cells with compound C16 mitigates the effects of a high-fat diet [143]. In congestive heart failure, PKR hyperactivation also occurs, which greatly exacerbates the course of the disease by increasing the level of cardiomyocyte apoptosis and the secretion of pro-inflammatory and profibrotic factors [144]. Thus, PKR dysfunction is implicated in the pathophysiology of cardiovascular diseases, and the use of PKR modulators can mitigate the course of these diseases.

Abnormal PKR function has also been implicated in the pathophysiology of non-hereditary PVOD, a condition that arises from the utilization of chemotherapeutic compounds, including cisplatin, bleomycin, and mitomycin C (MMC) [145]. Furthermore, MMC, in conjunction with the establishment of the characteristic clinical manifestation of PVOD, triggers the PKR branch of ISR signaling. Consequently, targeted therapy with the PKR inhibitor C16 or the ISR inhibitor ISRIB substantially mitigates the condition, while preventive treatment with ISRIB entirely reverses the progression of PVOD [145]. In the context of MMC-induced PVOD, activated ISR signaling contributes to the development of the disease by promoting pulmonary vascular remodeling and right ventricular hypertrophy through, among other mechanisms, the suppression of the synthesis of vascular endothelial cadherin and RAD51, adhere junction proteins [145]. With age, there is a decrease in the activity of protein phosphatase 1, which normally limits the activation of the ISR cascade, and an increase in the basal activity of ISR signaling. This ISR pathway malfunction underlies an exacerbation of MMC-induced PVOD in elderly patients [146]. Interestingly, the administration of ISRIB and C16 has been observed to alleviate even this complicated form of PVOD, thereby underscoring the profound involvement of ISR signaling in the development of PVOD pathophysiology. These findings offer a renewed perspective on the potential of ISR inhibitors to emerge as a novel therapeutic approach for the management of PVOD in the future [146].

#### 3.3.3. PKR and Inflammasome Response

In addition to its canonical role in eIF2α phosphorylation, PKR plays a key role in regulating innate immune responses, particularly through its interaction with inflammasome components and modulation of inflammatory gene expression.

PKR has been shown to physically interact with inflammasome-associated proteins such as NLRP1, NLRP3, NLRC4, and AIM2, facilitating inflammasome assembly and activation in various pathological contexts [147]. PKR also activates another inflammasome-associated component, NALP3. Pharmacological inhibition of PKR—via agents such as 2-aminopurine or C16, or genetic knockout—markedly attenuates inflammatory responses and diminishes inflammasome activity [147]. For instance, C16 has been shown to suppress inflammasome formation and enhance tissue repair (angiogenesis, collagen deposition) in models of diabetic wounds [148].

In atherosclerosis, ox-LDL has been shown to stimulate PKR activation indirectly via upregulation of the purinergic P2X7 receptor, which in turn enhances NLRP3-mediated inflammasome assembly and promotes vascular inflammation [149]. Similarly, PKR activation in pulmonary hypertension (PH) promotes inflammasome formation and the release of IL-1β and HMGB1, which in turn stimulate vascular smooth muscle cell proliferation, resulting in luminal narrowing [150]. Both genetic deletion of PKR and pharmacological inhibition (e.g., 2-aminopurine) have been shown to ameliorate PH pathology by suppressing inflammasome formation and pro-inflammatory secretion in vascular cells [150].

In a model of diabetic retinopathy, PKR activation induces NLRP3-mediated inflammasome assembly in endothelial cells. This effect is attenuated by both C16 and the Epac1 agonist 8-CPT-2′-O-Me-cAMP, which acts as a negative regulator of PKR [151]. On the other hand, PKM2, a glycolytic enzyme, functions as a positive regulator of PKR, promoting inflammasome formation. Increased activity of the PKM2 protein has been associated with an increased risk of vascular atherosclerosis. In diabetic atherosclerosis models, both the PKR inhibitor C16 and salvianolic acid A, which suppresses PKM2 activity, were shown to protect endothelial cells from high-glucose-induced damage [152].

These findings suggest that modulation of PKR activity affects both the ISR and the process of inflammasome formation. However, the majority of experimental studies exploring the role of PKR in vascular pathology consider these pathways separately, without addressing their potential interaction. This represents a limitation, as a more comprehensive analysis of PKR signaling is required to fully understand its diverse molecular functions. The available evidence supports the notion that PKR may serve as a regulatory node integrating the ISR and innate immune responses. Furthermore, the anti-inflammatory properties of PKR inhibitors are receiving growing attention, as several antioxidant compounds have been shown to influence PKR activity in vascular cells [148,153].

Beyond inflammasomes, PKR also contributes to broader inflammatory signaling. It can activate the NF-κB pathway by phosphorylating its inhibitor IκB, thereby inducing the expression of pro-inflammatory interferon genes [154,155,156]. Transcriptome profiling of EIF2AK2-overexpressing cells has shown that PKR regulates hundreds of genes, including those involved in antiviral responses, T-cell signaling, and innate immunity, further confirming its role as a broad immune modulator [157].

In summary, it is important to acknowledge that the mechanisms of action of PKR kinase are multidirectional and have been extensively described in a number of experimental articles. Furthermore, various compounds have been developed with a specific focus on modulating PKR activity, and other agents with other therapeutical applications were found to modulate PKR [153,158,159,160]. This extensive array of data and tools enables a thorough evaluation of PKR’s role in specific pathological conditions. However, a comprehensive assessment is absent in the majority of scientific papers. Consequently, PKR is frequently examined within the confines of ISR signaling or exclusively in relation to the STAT1 cascade or the NLRP3 signaling pathway. It is imperative to note that, given the multifaceted functions of PKR, which encompass the regulation of macrophage polarization towards the M1 phenotype and the stimulation of tumor neo-angiogenesis, a comprehensive and nuanced understanding of this kinase’s mechanisms of action necessitates consideration of the three aforementioned signaling pathways [132,161,162].

The case of PKR further reinforces the context-dependent nature of ISR signaling in vascular pathologies. PKR modulates diverse cellular processes—from proliferation and apoptosis to inflammasome activation and senescence—often with opposing outcomes depending on cell type and disease context. In vascular smooth muscle cells, PKR-mediated cell cycle arrest can be beneficial in preventing restenosis, yet in macrophages, the same pathway promotes pro-inflammatory polarization and atherosclerosis. Likewise, PKR-driven inflammasome activation may support host defense but simultaneously exacerbate vascular inflammation and tissue damage. These apparent contradictions underscore the central conclusion of this review: ISR kinases act as double-edged regulators whose effects are shaped by temporal, spatial, and cellular context. Therefore, future therapeutic strategies must move beyond simple inhibition or activation and aim for precise, tissue-specific ISR modulation tailored to disease mechanisms.

### 3.4. HRI

The heme-regulated inhibitor (HRI) kinase is encoded by the EIF2AK1 gene and is vital for erythroid cells. When intracellular concentrations of heme are sufficient, it binds to the HRI and blocks it, thereby allowing for globin translation. HRI deficiency leads to proteotoxicity caused by excessive globin synthesis. Another triggering mechanism for the HRI is mitochondrial stress [163]. Uncoupling of the mitochondrial electron transport chain activates the inner mitochondrial membrane-localized protease OMA1, which cleaves the intermembrane space-localized large form of DELE1 (L-DELE1) to the small form of DELE1 (S-DELE1) [164]. Under physiological conditions, L-DELE1 is readily imported into the mitochondria, where it is degraded by the matrix-localized, ATP-dependent serine protease, LONP1. In iron deficiency, import of L-DELE1 through the inner mitochondrial membrane is blocked. This traps L-DELE1 in the mitochondrial intermembrane space where it is processed into the S-DELE1 by the cleavage site of the OMA1 protein [165]. S-DELE1 can enter the cytoplasm, where it activates the HRI by binding to it. [164]. Additionally, the HRI can indirectly trigger a distinct type of mitophagy via ISR signaling that is mechanistically distinct from ubiquitin-mediated mitophagy [166]. Mitochondrial DNA breaks also lead to DELE1-HRI-dependent activation of the ISR pathway [167]. Thus, the HRI kinase is a sensor for both heme deficiency and mitochondrial stress.

Although the HRI was the first kinase identified for eIF2α phosphorylation [163], relatively little attention has been given to studying its function compared to PKR, PERK, and GCN2 kinases. This is largely because the HRI’s role in mitochondrial maintenance was only recently described. For a long time, the HRI was considered exclusively a sensor of heme deficiency [163]. Similarly, only recently have previously known compounds been studied for their modulatory effect on the HRI kinase, and new HRI modulators have been developed. The following compounds are among the known HRI activators: uncoupler agents carbonyl cyanide m-chlorophenyl hydrazone (CCCP) [168] and 1-(Benzo[d][1,2,3]thiadiazol-6-yl)-3-(3,4-dichlorophenyl)urea [169]; the ATP synthase blocker oligomycin [170]; nucleoside mimetic compounds 0357 and 3610 [171]; the phosphodiesterase 3 inhibitor, parogrelil, and G protein-coupled receptor 119 agonist, MBX-2982 [172]. Several HRI kinase inhibitor molecules have also been developed [173]. Descriptions of the presented HRI modulators are scarce in the literature. However, since the discovery of the HRI’s role in maintaining mitochondrial homeostasis, interest in the HRI has gained new ground in scientific research. Therefore, we believe that, in the coming years, we can expect a rapid increase in research articles describing the therapeutic use of HRI modulators.

A recent study by Qin et al. has confirmed the involvement of the HRI kinase in maintaining mitochondrial homeostasis in endothelial cells [174]. Using human umbilical vein endothelial cells, the authors demonstrated that a deficiency in GTP-binding protein 3, an enzyme involved in tRNA modification, leads to inflammation and reduced angiogenesis mediated by the HRI-ISR pathway [174]. Knocking down the HRI using siRNA reversed these negative effects, including rescuing tube formation and endothelial cell proliferation [174]. A similar effect to that of HRI siRNA was observed when endothelial cells were treated with ISRIB, an ISR signaling inhibitor [174]. Upcin et al. used an ex vivo aortic ring angiogenesis assay and tumor organoid models involving the co-culture of endothelial cells, macrophages, fibroblasts, and human melanoma or breast cancer cells to demonstrate that the pharmacological activation of the HRI inhibits angiogenesis [175]. Thus, studying the HRI in the context of normal and pathological vascular physiology is of great interest but requires more scientific work to develop a hypothesis about the HRI’s role in vascular cells.

**Table 1:** Pharmacological characteristics of ISR modulators and their applications in vascular diseases.

ISR Component	Modulator	Effect on ISR Pathway	Side Effects	Off Targets	Pathology	Pharmacological Effect
PERK	GSK2606414	Inhibition	Pancreas toxicity [176,177,178,179] Body weight loss [180,181,182] Hyperglycemia [181]	At a concentration of less than 1 μM: RIPK1 [182] c-kit [183,184] Aurora B kinase [184] BRK [184] MLK2/MAP3K10 [184] c-MER [184] DDR2 [184] MLCK2/MYLK2 [184] IKKe/IKBKE [184] Concentration of 1 μM: IKKe/IKBKE [184] TRKC [184] MLK3/MAP3K11 [184] RET [184] LCK [184] NEK4 [184] KHS/MAP4K5 [184] MLK1/MAP3K9 [184] TRKA [184] AXL [184] TRKB [184] YES/YES1 [184] WNK2 [184]	Atherosclerosis	Beneficial [69,73]
					Hypertension	Beneficial [74]
					Restenosis	Beneficial [82,83]
					Inflammation	Beneficial [83]
					Tumor growth	Beneficial [63,64]
					Thrombosis	Beneficial [82]
	GSK2656157	Inhibition	No toxic effects on heart, liver, kidney and lung tissues [185]	RIPK1 [182] HRI [186] PKR [186] GCN2 [186,187]	Diabetic cardiomyopathy	Detrimental [62]
					Tumor growth	Beneficial [66]
	CCT020312	Activation	No toxic effects on liver and kidney [188]	no data	Hypertension	Detrimental [74]
					Tumor growth	Detrimental [63,64]
GCN2	Halofuginone	Activation	Skin and eyes irritation, acute toxicity at high doses [189]	TGF-β1/Smad 3 signaling [127,190,191,192,193]	Inflammation	Beneficial [124,125,127]
					PH	Beneficial [126,127]
					Restenosis	Beneficial [128]
					Tumor growth	Beneficial [129]
PKR	C16	Inhibition	no data	CDK2/CDK5 [194]	Hypertension	Beneficial [142]
					Inflammation	Beneficial [142,143,148,151]
					PVOD	Beneficial [145,146]
					Atherosclerosis	Beneficial [152]
	2-aminopurine	Inhibition	Mutagenic effects, irritation to eyes, skin, and respiratory tract [195]	p53 signaling [196] DNA synthesis [197]	Senescence	Beneficial [133]
					Inflammation	Beneficial [147,150]
					PH	Beneficial [150]
Phosphorylation of eIF2α	ISRIB	Inhibition	No overt signs of toxicity [176,198]	no data	PVOD	Beneficial [145,146]
	Salubrinal	Activation	No overt signs of toxicity [199,200,201]	Bcl-2 [202]	Atherosclerosis	Beneficial [71,75,84]
					PVOD	Beneficial [90]

Effects are color-coded: green denotes beneficial effects, red denotes detrimental effects.

## 4. Conclusions

In this review, we have comprehensively examined the role of integrated stress response signaling in vascular physiology. Mounting evidence supports the notion that ISR activation—particularly through PERK, GCN2, and PKR—is essential for key aspects of vascular function, including angiogenic signaling, stress adaptation, and maintenance of endothelial integrity. At the same time, sustained ISR activation contributes to vascular inflammation, apoptosis, and pathological neovascularization, particularly in the context of tumor progression and atherosclerosis.

**Figure 3:**
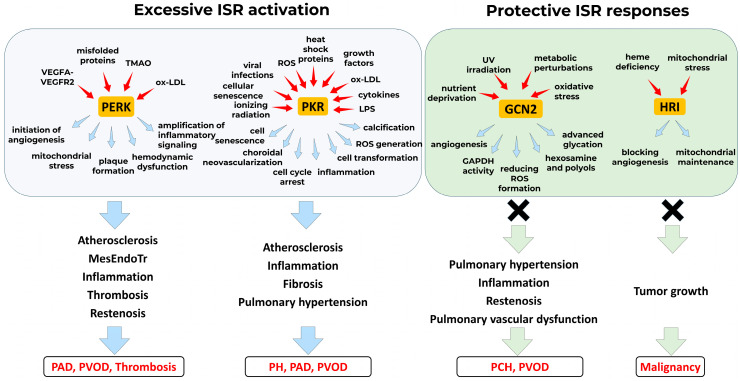
Context-dependent roles of ISR kinases in vascular and cancer-related pathology. The schema contrasts outcomes of excessive ISR activation (**left**) versus protective ISR responses (**right**). In the “excessive” arm, PERK and PKR are activated by stressors relevant to vascular disease (e.g., misfolded proteins/ER stress, TMAO, ox-LDL, viral infection, ROS/heat-shock responses, cytokines and LPS), promoting pro-atherogenic and pro-inflammatory processes including initiation of angiogenesis, mitochondrial stress, plaque formation, hemodynamic dysfunction, inflammatory signal amplification, calcification, ROS generation, senescence/cell-cycle arrest, and cell transformation; these changes are associated with atherosclerosis, EndoMT, inflammation, thrombosis, restenosis, and pulmonary hypertension, culminating in clinical entities such as PAD/PVOD (and thrombosis) and PH/PAD/PVOD. In the “protective” arm, GCN2 and HRI support adaptive programs in response to nutrient deprivation, UV/metabolic/oxidative stress, heme deficiency, and mitochondrial stress (e.g., redox control, metabolic adaptation, mitochondrial maintenance, and context-dependent angiogenesis control).

While PERK and PKR are primarily associated with the detrimental consequences of sustained ISR signaling, GCN2 appears to mediate more protective responses. Loss-of-function biallelic mutations in the EIF2AK4 gene, which encodes GCN2, lead to severe pulmonary vascular remodeling and the development of PVOD or PCH. Conversely, in preclinical models of pulmonary hypertension, pharmacological activation of GCN2 by halofuginone has been shown to attenuate vascular remodeling, oxidative stress, and inflammation—supporting a protective role in this pathological setting [94,126,127]. Furthermore, GCN2 protein levels are markedly reduced in the pulmonary endothelium of patients with pulmonary fibrosis and associated pulmonary hypertension, independent of PH severity. In vivo, genetic or pharmacological disruption of GCN2 exacerbates lung injury, remodeling, and PH in rats. This effect has been mechanistically linked to dysregulated TGF-β and IL-6 signaling, endothelial dysfunction, hyperpermeability, and a propensity for endothelial-to-mesenchymal transition [118]. In addition, GCN2 signaling promotes endothelial cell survival, upregulates the pro-angiogenic factor VEGFA, and enhances cellular resilience to metabolic stress via the ATF4 pathway [93]. These context-dependent roles of ISR kinases are depicted in Figure 3.

The majority of experimental work relies on pharmacological modulation of the ISR to dissect its functional consequences. Several ISR modulators have shown efficacy in preclinical models by suppressing endothelial apoptosis, vascular inflammation, and atherosclerotic progression. This suggests that selective ISR modulators may hold promise as novel therapeutic agents for vascular diseases. However, the pharmacokinetics, bioavailability, and long-term safety of these compounds remain poorly characterized. Notably, both ISR inhibitors (e.g., C16, 2-aminopurine, GSK2606414, GSK2656157) and activators (e.g., salubrinal, halofuginone) have demonstrated beneficial effects—underscoring the need for context-dependent application of ISR modulators tailored to specific vascular conditions and disease stages.

Furthermore, the molecular mechanisms underlying the dual effects of ISR modulation are still not fully understood. These knowledge gaps, combined with the paucity of in vivo studies, currently limit the translational potential of ISR-targeted therapies and hinder their progression into clinical trials.

## 5. Future Directions

While the preclinical evidence for targeting the ISR in vascular diseases is compelling, several critical gaps hinder its clinical translation. Future research should prioritize the following directions:

- Precision Therapeutics and Targeted Delivery

Current ISR inhibitors or activators (e.g., GSK2606414, halofuginone, C16) often exhibit broad activity across multiple eIF2α kinases and cell types. This lack of specificity raises significant concerns regarding on-target toxicity, including pancreatic injury [66,176,181,203].

- Overcoming these limitations will require two complementary strategies. First, the rational design of next-generation ISR modulators—including improved ISRIB analogues and novel allosteric compounds—should prioritize enhanced selectivity for specific ISR branches (e.g., PERK- vs. GCN2-specific) and optimized pharmacokinetic and safety profiles. Second, advances in delivery technologies, such as lipid nanoparticles, liposomes, and other nanocarriers, offer promising platforms for transient, non-genomic, and cell-specific modulation of ISR pathways. These clinically validated systems—already applied in mRNA vaccines and siRNA therapies—can be adapted to deliver ISR modulators (e.g., mRNA-encoded dominant-negative constructs or siRNAs targeting ISR effectors) to vascular smooth muscle cells, endothelial cells, or lesion macrophages, thereby minimizing systemic exposure and reducing off-target effects.
- Deciphering Cell Type-Specific ISR Dynamics In Vivo. The functional outcome of ISR activation is highly context-dependent, varying by cell type (endothelial cells, VSMCs, macrophages), disease stage, and metabolic milieu. Leveraging single-cell RNA seq and other multi-omics technologies (transcriptomics, proteomics) on human vascular tissues and advanced animal models will be essential to map these heterogeneous responses and identify the most therapeutically vulnerable cellular targets.
- Elucidating the Switch from an Adaptive to Maladaptive ISR. A fundamental unanswered question is what molecular mechanisms determine whether ISR signaling promotes cell survival or initiates apoptosis in vascular cells. Systematic studies combining genetic screening with temporal phospho-proteomics and metabolomics are needed to identify the critical checkpoints that dictate this decision. A paramount challenge is to map the “therapeutic windows” for ISR modulation across different vascular diseases. Computational modeling of ISR network dynamics in specific vascular cell types could further predict the tipping point between adaptive and maladaptive outcomes, guiding the timing and choice of intervention.
- Bridging the Translational Gap. Despite promising preclinical findings, in vivo data on the pharmacokinetics, long-term safety, and therapeutic efficacy of ISR modulators in chronic vascular disease contexts remain limited. Moving beyond proof-of-concept will require robust, investigational new drug (IND)-enabling studies, including dose–response modeling, toxicological profiling, and biomarker-guided efficacy assessment. Expanding ISR modulation to conditions such as age-related vascular dysfunction is especially compelling, given the well-established links between ISR, senescence, and vascular aging.
- Integrating the ISR with Vascular Immunology. The crosstalk between the ISR and inflammasome activation, particularly via PKR, in endothelial cells and macrophages is a potent driver of pathology. A more integrated understanding of this axis could reveal novel combinatorial strategies to simultaneously dampen inflammatory and proteotoxic stress in diseases like atherosclerosis and pulmonary hypertension.

Addressing these challenges will require a concerted interdisciplinary effort. However, the reward is the potential to translate the intricate biology of the ISR into a new class of mechanism-based therapies for vascular diseases that currently lack effective causal treatments.

Taken together, ISR signaling represents a central regulatory pathway in both normal and pathological vascular biology. While pharmacological modulation of this pathway holds therapeutic promise, further mechanistic insight and translational validation are essential for its clinical application.

## Data Availability

No new data were created or analyzed in this study.

## Supplementary Materials

The following supporting information can be downloaded at: https://www.mdpi.com/article/10.3390/cells15010002/s1. Abbreviations list.
