# Supplementary material for: Integrated Stress Response (ISR) Modulators in Vascular Diseases
Source: Cells. 2025 Dec 19;15(1):2. doi: 10.3390/cells15010002 (PMC12786323; doi:10.3390/cells15010002)
Supplement: Supplementary file 1 [file cells-15-00002-s001.zip › cells-4039073-supplementary.pdf]

Abbreviations List

1. AIM2 – Absent In Melanoma 2
2. Ang II – angiotensin II
3. ATF3/4/5/6 – Activating Transcription Factor 3/4/5/6
4. ATG5/12 – Autophagy Related 5/12
5. ATFS-1 – Activating Transcription Factor associated with Stress-1
6. bZIP – basic Leucine Zipper
7. CAFs – cancer-associated fibroblasts
8. CaMKII – Ca^{2+}/Calmodulin-dependent protein Kinase II
9. CCCP – carbonyl cyanide m-chlorophenyl hydrazone
10. cGAS – cyclic GMP-AMP Synthase
11. CHOP (DDIT3) – C/EBP Homologous Protein (DNA Damage Inducible Transcript 3)
12. CNPY2 – canopy FGF signaling regulator 2
13. CReP (PPP1R15B) – Constitutive Repressor of eIF2α Phosphorylation
14. Crc – cryptocephal
15. DELE1 – DAP3 Binding Cell Death Enhancer 1
16. dsDNA – double-stranded DNA
17. Edn1 – Endothelin-1
18. eIF1/1A/2/2B/3/4/5/5B – eukaryotic Initiation Factor 1/1A/2/2B/3/4/5/5B
19. EIF2AK1/2/3/4 – Eukaryotic Translation Initiation Factor 2 Alpha Kinase 1/2/3/4
20. EndoMT – endothelial-to-mesenchymal transition
21. Epac1 – Exchange protein directly activated by cAMP 1
22. ER – Endoplasmic Reticulum
23. ERK1/2 – Extracellular signal-Regulated Kinases 1/2
24. FAM69C – Family With Sequence Similarity 69 Member C
25. FBXO22 – F-Box Protein 22
26. FGF21 – Fibroblast Growth Factor 21

27. GADD34 (PPP1R15A) – Growth Arrest and DNA Damage-inducible protein 34
28. GCN2 (EIF2AK4) – General Control Nonderepressible 2
29. GCN4p – General Control Nonderepressible 4 protein (дрожжи)
30. GDP/GTP – Guanosine Di-/Tri-Phosphate
31. GRP78 (BiP/HSPA5) – Glucose-Regulated Protein 78 (Binding Immunoglobulin Protein / Heat Shock Protein Family A (Hsp70) Member 5)
32. HIF-1α – Hypoxia-Inducible Factor 1-alpha
33. HMGB1 – High Mobility Group Box 1
34. HRI (EIF2AK1) – Heme-Regulated Inhibitor
35. IL-1β/6 – Interleukin-1 beta / 6
36. IND – Investigational New Drug
37. IRE1α – Inositol-requiring enzyme 1 alpha
38. ISR – Integrated Stress Response
39. ISRIB – Integrated Stress Response Inhibitor
40. JNK – c-Jun N-terminal Kinase
41. KEAP1 – Kelch-like ECH-associated protein 1
42. LC3 – Microtubule-associated protein 1A/1B-light chain 3
43. LDL – Low-Density Lipoprotein
44. LONP1 – Lon Peptidase 1
45. LOX-1 – Lectin-like Oxidized LDL receptor-1
46. Lp(a) – Lipoprotein(a)
47. LPS – Lipopolysaccharide
48. MAPK – Mitogen-Activated Protein Kinase
49. MARK2 – Microtubule Affinity Regulating Kinase 2
50. MCT-1/DENR – MCT-1/ Density-regulated protein
51. Met-tRNAi^Met^ – initiator methionyl-transfer RNA
52. MMC – mitomycin C
53. mRNA – messenger RNA

54. mTORC1 – mechanistic Target Of Rapamycin Complex 1
55. NALP3/NLRP1/3/NLRC4 – NACHT, LRR and PYD domains-containing protein 3 / NLR Family Pyrin Domain Containing 1/3 / NLR Family CARD Domain Containing 4
56. NO – nitric oxide
57. NOX2/4 – NADPH Oxidase 2/4
58. NRF2 – Nuclear factor erythroid 2–related factor 2
59. OMA1 – OMA1 Zinc Metallopeptidase
60. ox-LDL – oxidized Low-Density Lipoprotein
61. PAD – Peripheral Artery Disease
62. PAH – Pulmonary Arterial Hypertension
63. PAM – Peptidyl-glycine α-Amidating Monooxygenase
64. PCH – Pulmonary Capillary Hemangiomatosis
65. PDGF – Platelet-Derived Growth Factor
66. PERK (EIF2AK3) – Protein kinase RNA-like Endoplasmic Reticulum Kinase
67. PH – Pulmonary Hypertension
68. PI3K/Akt – Phosphoinositide 3-kinase / Protein kinase B
69. PKM2 – Pyruvate Kinase M2
70. PKR (EIF2AK2) – Protein Kinase R
71. PKZ – Protein kinase containing Z-DNA binding domains
72. PLCγ – Phospholipase C gamma
73. PP1/PP1c – Protein Phosphatase 1 / catalytic subunit
74. p27^kip1^ – cyclin-dependent kinase inhibitor 1B
75. PVOD – Pulmonary Veno-Occlusive Disease
76. RAD51 – RAD51 Recombinase
77. RAGE – Receptor for Advanced Glycation Endproducts
78. RIPK1 – Receptor-Interacting Serine/Threonine-Protein Kinase 1
79. RNA – Ribonucleic Acid

80. ROS – Reactive Oxygen Species
81. siRNA – small interfering RNA
82. SIRT1 – Sirtuin 1
83. STING – Stimulator of Interferon Genes
84. TC – Ternary Complex
85. TGF-β1 – Transforming Growth Factor beta 1
86. TMAO – Trimethylamine-N-oxide
87. TMT-LC-MS – Tandem Mass Tag labeling – Liquid Chromatography – Mass Spectrometry
88. TNF-α – Tumor Necrosis Factor alpha
89. TRAP1 – TNF Receptor Associated Protein 1
90. TWIST – Twist Family BHLH Transcription Factor
91. VCAM-1 – Vascular Cell Adhesion Molecule-1
92. VEGF(A/B) – Vascular Endothelial Growth Factor (A/B)
93. VEGFR2 – Vascular Endothelial Growth Factor Receptor 2
94. VSMCs – Vascular Smooth Muscle Cells
95. vWF – von Willebrand Factor
96. ZO-1 – Zonula Occludens-1
